#  Neonatal Perineal Tear: A Rare Birth Injury 

**Published:** 2016-01-01

**Authors:** Irom Keshorjit Singh

**Affiliations:** Department of Surgery (Pediatric Surgery), Regional Institute of Medical Sciences, Imphal, India

**Dear Sir**

Birth injury is an important cause of neonatal morbidity and mortality. It is more common when the delivery is conducted by untrained personals [1]. Birth injuries are more prone to develop in babies with macrosomia, fetal organomegaly, mass lesions, prematurity, protracted labor, precipitous delivery, breech presentation and cephalopelvic disproportion [2]. 

A (2.8 kg) 2-day-old female baby was brought to the hospital after delivery conducted by the local traditional healers. The baby was in sepsis with a perineal tear extending from the vagina/vestibule to the rectum running deep to the perineum about 2cms with devitalised tissues of peri-anal margin and vulva (Fig. 1). On careful evaluation of the history, delivery was assisted by pulling out the baby with thumb in anus and fingers around the buttock (evident by the presence of abrasions, cellulitis and maceration of the skin in the buttock) and vulva resulting in the injury. The blood culture and wound swab showed growth of E coli. The baby was put on cefotaxime, amikacin and metronidazole. Thorough wound irrigation with debridement of devitalised tissues was done. The rectal mucosa and vaginal mucosa were repaired with interrupted vicryl 5–0. The perineal body and muscles were repaired with vicryl 4–0, and perineal skin with vicryl 5–0. Loop sigmoid colostomy and on table distal stoma saline wash was done to clear the meconium from the rectum. Colostomy was closed after 6 weeks after the adequate anal dilatation was achieved. At 12 months follow up, the stool frequency is 1 – 2 per day and continent.

**Figure:**
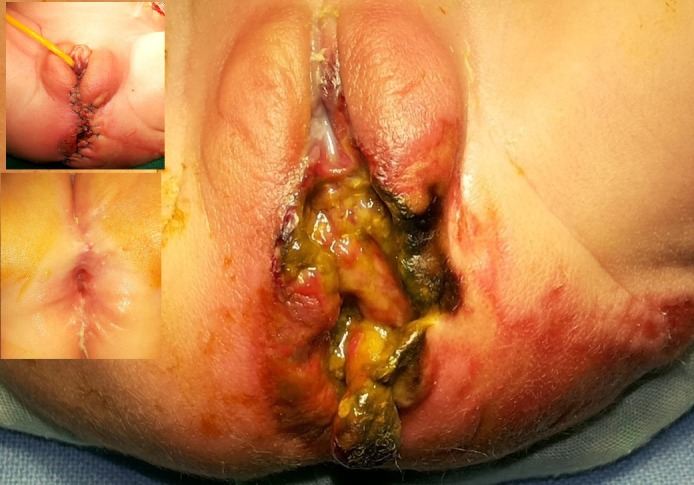
Figure 1: Contaminated perineal wound with gangrenous vulval margins, devitalised and gangrenous peri-anal tissue with surrounding cellulitis. Insets show repaired and healed lesions.

Birth injuries are more common and severe in developing countries because of lack of available trained personnel [1]. Very few cases of perineal / recto-vaginal injury during different modes of delivery have been described by authors with different mechanisms of injury. Caesarean section remains the method of choice to avoid these problems, but injuries have been reported due to the technique rather than the mode of delivery [1]. Different mechanisms of injury to the perineum of the neonates has been described in the literature such as repeated maternal vaginal examinations, fetal scalp electrode application during breech delivery, blunt finger dissection of the endometrium during caesarean section, manual attempt to rotate a fetus in an unfavourable fetal lie, during artificial rupture of membrane [1,3-5]. We are reporting a new mechanism of neonatal perineal injury caused by assisting the delivery in a breech presentation which has not been described before. Management of the wound as described in the literature includes conservative management for low grade injuries, multilayer primary repair with or without diverting colostomy [1,3-5]. In the present case, covering colostomy was needed along with thorough wound irrigation, debridement with primary repair as the case was reported late with contaminated wound.

## Footnotes

**Source of Support:** Nil

**Conflict of Interest:** None
